# Advanced Bioluminescence System for In Vivo Imaging with Brighter and Red-Shifted Light Emission

**DOI:** 10.3390/ijms21186538

**Published:** 2020-09-07

**Authors:** Mizuki Endo, Takeaki Ozawa

**Affiliations:** Department of Chemistry, School of Science, The University of Tokyo, 7-3-1 Hongo, Bunkyo-ku, Tokyo 113-0033, Japan; endo@chem.s.u-tokyo.ac.jp

**Keywords:** bioluminescence, luciferase, luciferin, bioluminescence resonance energy transfer

## Abstract

In vivo bioluminescence imaging (BLI), which is based on luminescence emitted by the luciferase–luciferin reaction, has enabled continuous monitoring of various biochemical processes in living animals. Bright luminescence with a high signal-to-background ratio, ideally red or near-infrared light as the emission maximum, is necessary for in vivo animal experiments. Various attempts have been undertaken to achieve this goal, including genetic engineering of luciferase, chemical modulation of luciferin, and utilization of bioluminescence resonance energy transfer (BRET). In this review, we overview a recent advance in the development of a bioluminescence system for in vivo BLI. We also specifically examine the improvement in bioluminescence intensity by mutagenic or chemical modulation on several beetle and marine luciferase bioluminescence systems. We further describe that intramolecular BRET enhances luminescence emission, with recent attempts for the development of red-shifted bioluminescence system, showing great potency in in vivo BLI. Perspectives for future improvement of bioluminescence systems are discussed.

## 1. Introduction

Quantitative monitoring of individual biomolecular reactions and cellular behaviors is necessary to elucidate the dynamic and complicated functions of biological phenomena. In vivo bioluminescence imaging (BLI) has enabled visualization of biological processes in intact living organisms, providing abundant quantitative, spatiotemporal information that is far beyond the reach of conventional in vitro assays. BLI is based on the catalytic activity of luciferase enzymes, which oxidize the substrate called luciferin to generate an excited-state molecule that emits bioluminescence. Unlike the fluorescence imaging technique, BLI requires no external excitation light that might cause background fluorescence or phototoxicity in analyzed samples. Therefore, one can achieve exquisitely sensitive imaging in opaque heterogeneous tissues. Consequently, BLI has enabled in vivo monitoring of various biological processes such as tumor growth, cancer metastasis, and bacterial infection [1].

**Figure 1:**
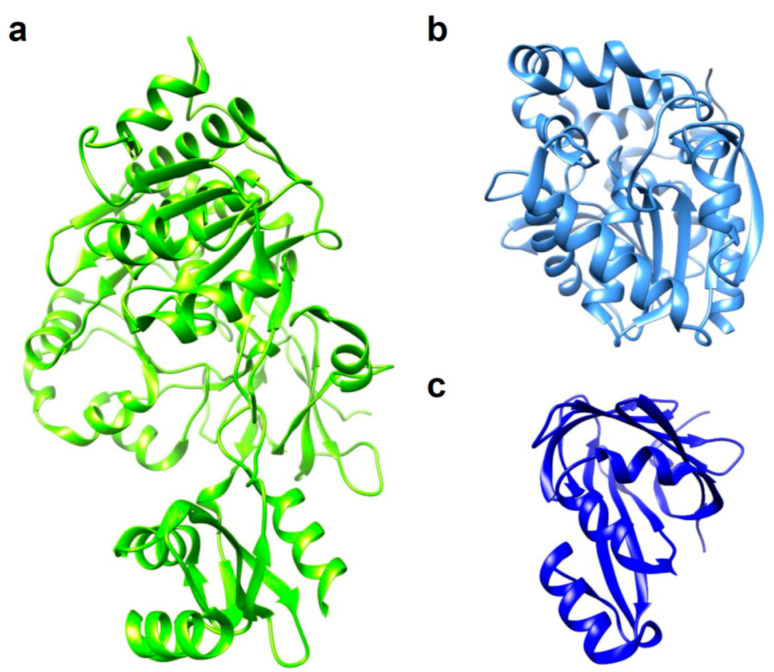
3D structures of luciferases used in in vivo bioluminescence imaging (BLI). (**a**) *Photinus pyralis* firefly luciferase (FLuc); PDB ID: 5DVQ. (**b**) *Renilla reniformis* luciferase (RLuc); PDB ID: 2PSD. (**c**) NanoLuc luciferase (NLuc); PDB ID: 5IBO.

**Figure 2:**
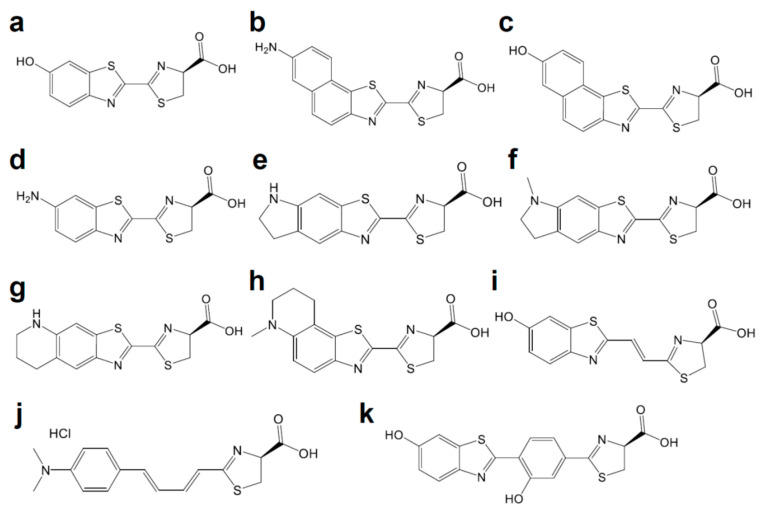
Chemical structures of d-luciferin and its red-shifted derivatives: (**a**) d-luciferin, (**b**) NH_2_-NpLH_2_, (**c**) OH-NpLH_2_, (**d**) 6′-aminoluciferin, (**e**) CycLuc1, (**f**) CycLuc2, (**g**) CycLuc7, (**h**) CycLuc10 (**i**) Infra-luciferin, (**j**) Akalumine-HCl, (**k**) PhOH-Luc.

**Figure 3:**
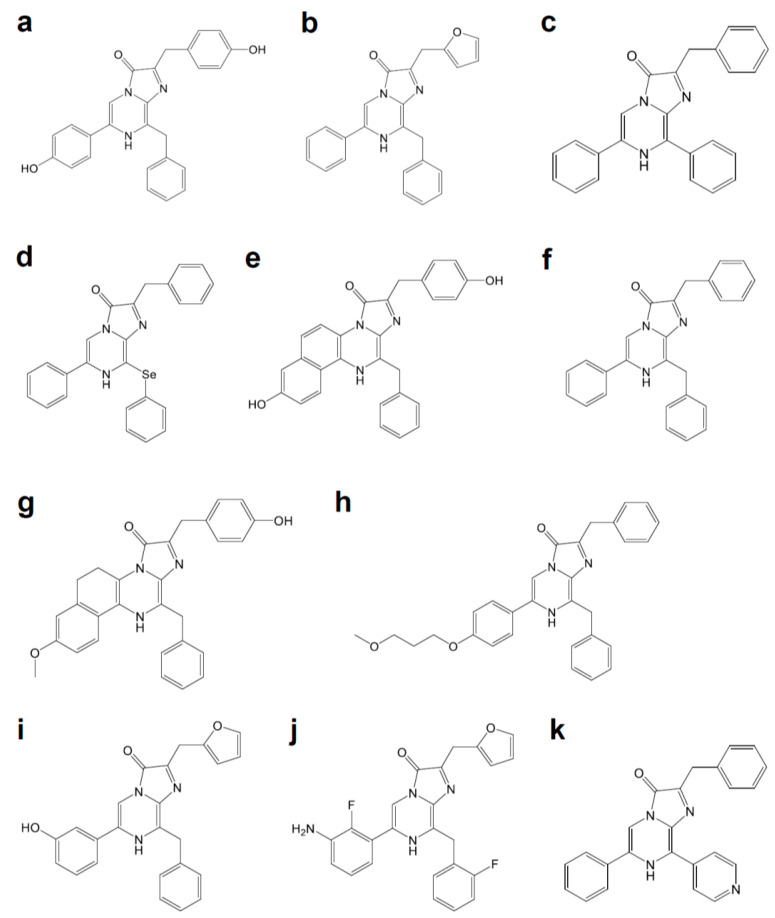
Coelenterazine derivatives for brighter or red-shifted bioluminescence systems: (**a**) Coelenterazine, (**b**) Furimazine, (**c**) Diphenylterazine, (**d**) Selenoterazine, (**e**) Coelenterazine-v, (**f**) DeepBlueC, (**g**) methoxy-eCoelenterazine, (**h**) BBlue2.3, (**i**) Hydrofurimazine, (**j**) Fluorofurimazine, (**k**) 8pyDTZ.

Luciferases used in BLI are derived from diverse species that include beetles, sea pansy, copepods, and deep-sea shrimp. The corresponding luciferins hold distinct chemical structures: firefly luciferase (FLuc, Figure 1a) requires d-luciferin (Figure 2a), while marine *Renilla* luciferase (RLuc, Figure 1b) and *Oplophorus* luciferase (OLuc) use coelenterazine (Figure 3b). All luciferins require chemical reactions to emit bioluminescence: the oxidation of luciferins by luciferases to yield excited-state oxyluciferin, and relaxation to the ground state with photon emission. Despite their similarity, the color and intensity of the emitted bioluminescence and the dependency on pH and other molecules (e.g., ATP) differ among luciferase–luciferin pairs. Therefore, the selection of appropriate luciferase–luciferin pairs according to the biological process of interest is crucially important for BLI.

Typical natural luciferases emit weak bioluminescence with wavelengths shorter than 650 nm. Therefore, the major obstacles hindering the wider use of in vivo BLI have been the absorption and scattering of emitted bioluminescence by the host tissue. To overcome the defects, many studies have been conducted to yield brighter, red-shifted bioluminescence systems by genetic or chemical engineering. This review briefly explains recent advances in the development of engineered bioluminescence systems. To elucidate the improvements of bioluminescence intensity, we first specifically describe the mutagenic engineering on FLuc, RLuc, and OLuc. Then, we introduce another approach for enhancing bioluminescence intensity using fluorescent proteins, which is based on bioluminescence resonance energy transfer (BRET). Regarding wavelength engineering, we introduce both genetic luciferase manipulation and chemical luciferin engineering. We offer conclusions and perspectives for the future improvement of bioluminescence systems.

## 2. Development of a Brighter Bioluminescence System

The emitted bioluminescence brightness is affected by the stability of the protein, or by the parameters of the catalytic reaction, such as the quantum yield, catalytic rate, and sensitivity to product inhibition [2]. Therefore, the engineering of luciferase enzymes or luciferin substrates is a rational approach to improve the brightness of bioluminescence systems (Table 1). Intramolecular BRET has also enabled the striking enhancement of bioluminescence intensity (Table 2). This section will specifically assess both approaches to develop a brighter bioluminescence system.

### 2.1. Engineering on Luciferase and Luciferin

Luciferase isolated from the North American firefly *Photinus pyralis*, FLuc (61 kDa, Figure 1a), is the most extensively investigated bioluminescence enzyme. FLuc catalyzes a two-step reaction that results in the oxidation of d-luciferin (Figure 2a) with ATP, Mg^2+^ and oxygen, which yields electronically excited oxyluciferin (OxyLH_2_). The accompanying bioluminescence emission peaks at 530–635 nm (pH-dependent), with a quantum yield at 0.48 [53,54]. However, BLI using native FLuc entails several shortcomings that include low thermostability and limited intracellular luciferin availability [55]. For example, the half-life of FLuc is less than 10 min at 35 °C [56]. The crystal structure of FLuc shows that the enzyme consists of the N-domain (1–436 a.a.), which is composed of subdomains A (1–190 a.a.) and B (191–436 a.a.), and C domain (443–544 a.a.) [57]. Because the middle subdomain B is less stable, it is the bottleneck that determines the stability of the whole protein [58]. Various mutations in subdomain B have been reported to improve FLuc thermostability, including T214A, A215L, I232A, F295L, and E354K [3,56,59]. The combination of 5 mutations improved FLuc thermostability up to a half-life of 11.5 hrs at 37 °C (Mutant E). Engineering of the protein surface is a general approach for enhancing protein stability [60]. Indeed, mutagenesis on solvent-exposed hydrophobic amino acids in FLuc (x5 FLuc; F14R, L35Q, V182K, I232K, and F465R) enhanced thermostability up to 45 °C with higher pH-tolerance [4]. With the help of those findings, 12 mutations to FLuc (x12 FLuc; F14R, L35Q, A105V, V182K, T214C, I232K, D234G, F295L, E354R, D357Y, S420T, and F465R) further improved the thermostability, which has a half-life of 15 min at 55 °C [6]. Thermostabilizing mutations indeed increase the light output intensity, as seen in the mutant called x5g (F14R, L35Q, V182K, I232K, V241I, G246A, F250S, and F465R) that exhibited 3.69-fold brighter luminescence than FLuc, which enabled in vivo bioluminescence imaging in the striatum of the mouse brain [5]. The increasing affinity and turnover rate for the ATP and d-luciferin substrates overcomes the low light output due to the limited availability of intracellular d-luciferin [61]. For example, mutation of Ile423 to Leu and Met remarkably increased the turnover rates. Mutations on Ile423 may affect the nearby hydrogen bonding between the carboxyl group of Asp422 and the hydroxyl group of AMP [62]. D436G mutation increased the substrate affinity for ATP and d-luciferin, which may because Asp436 inhibits substrate entry to a nearby cavity for ATP [63]. The increase of substrate affinity via L530R mutation might be due to Lys529, which is postulated to form an active site. By combining the thermostabilizing mutations (T214A, A215L, I232A, F295L, and E354K) and catalytic activity improving mutations (LGR; I423L, D436G, and L530R), a novel mutant named YY5 was generated, which has 14-fold higher *k*_cat_ than FLuc) [3].

Compared to d-luciferin-based ATP-dependent luciferases, luciferases using coelenterazine as their substrate (Figure 3a) present several important benefits as bioluminescent tags in BLI. They are ATP-independent and, in general, only require oxygen. *Renilla* luciferase (RLuc, Figure 1b), which comprises 311 amino acids, is a well-characterized coelenterazine-based luciferase with low molecular weight (37 kDa) and blue light (480 nm) emission. Although it is attractive for BLI, original RLuc is adversely affected by rapid inactivation, which has limited its biotechnological applications, especially in an in vivo model [21]. To overcome the defect, eight mutations (A55T, C124A, S130A, K136R, A143M, M185V, M253L, and S287L) were introduced, which are called RLuc. The RLuc8 showed a 4.3-fold enhancement in bioluminescence intensity compared to RLuc. Additional mutations (A123S, D154M, E155G, D162E, I163L, and V185L) of RLuc8 generated RLuc8.6-535, which emitted 6.0-fold higher luminescence than RLuc with a longer peak wavelength (535 nm) [22]. The bioluminescence from purified RLuc8.6-535 injected into the thigh musculature of the mouse (depth of ~ 1–2 mm) was detected by cooled CCD. Further improvements were achieved with BRET-based approaches, as introduced in the next section.

Recently, a novel luciferase called NanoLuc (NLuc, Figure 1c) was developed from a small subunit (19 kDa) of another coelenterazine-based OLuc [23]. First, nine mutations (A4E, Q11R, A33K, V44I, A54F, P115E, Q124K, Y138I, and N166R) generated variant C1A4E, which emits 88,000-fold brighter luminescence than the native OLuc small subunit. In parallel with further mutations that stabilize luciferase, a superior coelenterazine substrate was screened by modifications at the C-2, C-6, and C-8 positions of the imidazopyrazinone core. The coevolution approach produced NLuc and its selective substrate, named furimazine (Figure 3b). NLuc exhibited 150-fold greater activity than FLuc or RLuc, with its slow decay in luminescence (signal half-life > 2 h) and high physical thermal stability (up to 55 °C). The bright NLuc luminescence enabled subcellular BLI with 1–5 s exposure for image acquisition. It is particularly interesting that spontaneously interacting fragments of split NLuc were developed at the K_D_ for 700 pM. The resultant 11 amino acid peptide was used for quantifying proteins and monitoring protein trafficking [64,65]. Further mutation generated a brighter NLuc mutant called teLuc (D19S, D85N, and C164H), which emits 2.6-fold brighter bioluminescence than NLuc with diphenylterazine (Figure 3c), or yeLuc (F1L, A14D, L18Q, D19A, S28T, V27L, Q69R, R112Q, L142R, and C164S) which emits 11.5-fold brighter bioluminescence with selenoterazine (Figure 3d) [24].

### 2.2. BRET-Based Approaches

Főrster resonance energy transfer (RET) is an optical process that transfers energy from a donor to an adjacent acceptor fluorescent molecule [66]. In RET, the excess energy generated upon relaxation of the excited electron in the donor is transmitted to the acceptor as a virtual photon. The transfer is facilitated by the dipole-dipole couplings between the fluorophores, mostly depending on the spectral overlap and intermolecular distance. The efficiency of the energy transfer is dependent on the inverse sixth power of the fluorophore separation [67]. Notably, the distance between the donor and acceptor where the RET efficiency is 50% is called the Főrster distance, which normally falls into the nanometer range in fluorescence resonance energy transfer (FRET). Several marine organisms use unique mechanisms called BRET for bioluminescence emission, where the chemical energy liberated by the oxidization of the substrate is transferred from a luminescent donor to a fluorescent acceptor. The BRET efficiency also depends on intermolecular distance (1–10 nm) and relative orientation due to the similar RET mechanism [68,69].

**Figure 4:**
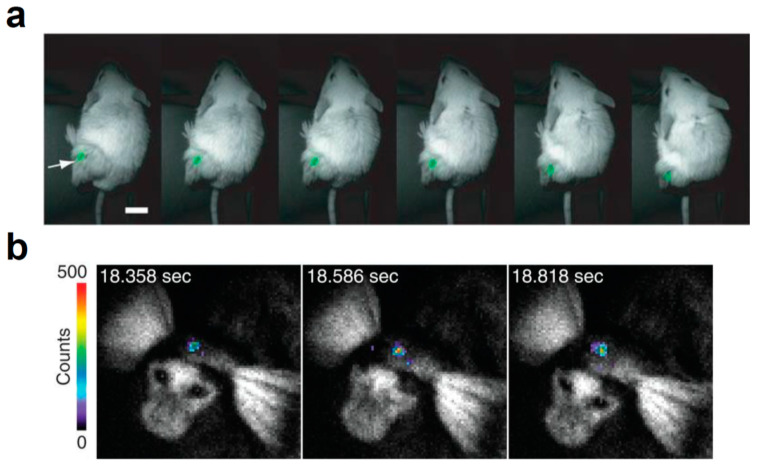
In vivo BLI with brighter or red-shifted bioluminescence systems. (**a**) Video-rate BLI of Nano-lantern-expressing tumor cells in an unshaved mouse; Scale bar, 1 cm. Reprinted with permission from ref. [34]. White arrow indicates the luminescence signal from Nano-lantern-expressing cells. Copyright 2012 Nature Publishing Group. (**b**) AkaBLI with 4-year-old female marmoset expressing AkaLuc in the right striatum 12 months after injection. Reprinted with permission from ref. [18]. Copyright 2018 The American Association for the Advancement of Science.

In luciferase engineering, BRET is typically used for modulating the wavelength of the emitted luminescence; however, the BRET modification sometimes yields enhanced luminescence from the engineered molecules. The first demonstration of intensity enhancement with intramolecular BRET was BAF-Y, an intermolecular BRET probe consisting of RLuc and EYFP [26]. BAF-Y exhibited a 7-fold increase in bioluminescence compared to RLuc. To further increase the luminescence intensity of BAF-Y, RLuc moiety was replaced with RLuc8, named eBAF-Y. eBAF-Y displayed 25-fold bioluminescence than RLuc, the spectrum of which showed a sharp peak at 525 nm. Further engineering of the fusion protein yielded a 2.9 times brighter protein, named Nano-lantern, when compared to eBAF-Y [34]. The increased bioluminescence intensity enabled BLI in freely moving unshaved mice (Figure 4a). A similar approach is also applicable to other luciferases, including the brightest luciferase: NLuc. The N-terminus of NLuc was fused with the C-terminus of EGFP, named GpNLuc LumiFluor. When provided with the corresponding luciferin, GpNLuc LumiFluor exhibited a 45-fold greater luminescence than Nano-lantern [40]. The strong bioluminescence allowed shorter exposure in in vivo BLI and ex vivo flow cytometry, thereby expanding the applicability of bioluminescence systems.

## 3. Development of Red-Shifted Bioluminescence System

Attempts at in vivo BLI have often been hampered by tissue scattering or absorption of light, leading to low signal-to-noise ratios in acquired images. The scattering in living tissue is mainly due to the Rayleigh scattering, which is caused by cell morphology or intracellular organelle [70,71]. The reduced coefficient, a quantitative parameter that describes wavelength or angular dependence of scattering, decreases with the increasing wavelength from 400 nm at least to 1200 nm in living tissue [72]. As for absorption, hemoglobin and melanin are the main chromophores that absorb light at the visible spectrum. Hemoglobin is a tetrameric protein that holds a heme group composed of a porphyrin ring and an iron ion, which is mainly contained in the red blood cells [73]. The heme iron reversibly binds and transports oxygen in the body. Hemoglobin strongly absorbs UV-light and visible light ranging from 400 nm to 800 nm, while the oxygen-bound oxyhemoglobin does so from 400 nm to 600 nm [74,75]. Melanins, such as eumelanin and pheomelanin, are heterogenous copolymers that act as pigments with potential biological functions, such as protection from solar radiation [76,77]. Their absorption spectrum ranges from 300 nm to 700 nm, with large absorption in the UV region [78]. In the far-red region, water also acts as a chromophore [79]. The vibrational overtone of the O-H bond yields absorption peaks around 970 nm and 1180 nm [80]. The so-called first biological window (NIR-I) spans the wavelength range from around 650 nm to 950 nm, where light is less absorbed by these chromophores in living tissue [81,82]. Therefore, for improving tissue penetration, the bioluminescence needs an emission peak around the red and near-infrared (NIR) regions, where photons are less absorbed or scattered by tissues.

To date, many efforts have been undertaken to generate red-shifted bioluminescence systems. This section presents descriptions of recent works with engineered luciferases and luciferins, which showed red or even NIR bioluminescence (Table 1), and red bioluminescence systems developed using a BRET-based approach (Table 2).

### 3.1. Engineering on Luciferase and Luciferin

The typical strategy for generating color variants of luciferase is to rationally induce mutations around the active pocket of the luciferase, or randomly induce mutations. The rational alteration of amino acids around the active pocket potentially modifies the chemical form of the excited luminescent substrate, leading to the color-shift in emitted bioluminescence. Beetle luciferases, including FLuc, use d-luciferin as a substrate to emit light ranging in color from green (530 nm) to red (635 nm) [54]. Although the color variation of chemiluminescence in OxyLH_2_, green (560 nm) and red (620 nm), is explained by keto-enol tautomers [83], both bioluminescence colors were considered to be from keto-constrained OxyLH_2_ [84]. This indicates that the color of firefly luminescence could be modulated within the keto-form, although the contribution of the enol-form is also proposed [85]. The spectroscopic study revealed that the polarity of the active site environment and the strength of covalent character of O8′···H bond between the excited phenolate anion of keto-form OxyLH_2_ and a protonated basic moiety in the active site modulates the light-color [86,87]. Of course, the hypothesis mentioned above may oversimplify the color-tuning mechanisms [88], indicating the limitation of rational site-directed mutagenesis.

Some mutagenetic attempts succeeded in generating a red-shifted FLuc bioluminescence system. An arginine residue (Arg218) near the active site is possibly involved in O8′···H interaction with OxyLH_2_. The site-directed mutagenesis on Arg218 of wild type FLuc (557 nm at pH 7.8) produced red-shifted variants, R218K (572 nm), R218Q (608 nm), and R218A (611 nm) [7]. Arg337 also locates near the binding site, whose mutation induced a red-shift in the emission spectra (R337K 595 nm, R337Q 594 nm). The other amino acid residues around the active sites were also subjected to site-directed mutagenesis. The obtained red-shifted mutants include H245A (604 nm), G315A (607 nm), T343A (617 nm), and A348V (610 nm) [8]. Mutations to amino acid residues not close to the active site may indirectly affect the molecular environment of substrate d-luciferin. The random mutagenesis approach identified red-shifting mutations S284T (615 nm) [9,10]. Structural analysis of similar mutants in *Luciola cruciata* luciferase (S286N) revealed the transient movement of nearby Ile288 toward the excited OxyLH_2_, which may be also the case with FLuc [12]. Sometimes, red-shifting mutations are accompanied by losses in thermal stability or catalytic activity. The losses can be alleviated by additional thermostabilizing mutations, as shown in the FLuc variant named Ppy RE9 was created by inducing red-shifting mutations (S284T, R330G), thermostabilizing mutations (T214A, A215L, I232A, F295L, E354I), and mutations that compensated accompanied losses in thermostability and enzyme activity in the red-shift (I351V and F465R). Ppy RE9 emits bioluminescence at 617 nm with ~11.5% of respective light intensity compared to FLuc [11].

The coevolution of luciferases and luciferins is a promising approach for generating an improved bioluminescence system for in vivo BLI. In an attempt to generate red-shifted variants of RLuc8, 22 residues putatively composing the active site were subjected to site-directed mutagenesis [22]. The mutations at eight of these candidate residues yielded detectable red-shifts in the emitted bioluminescence. Further engineering with random mutagenesis on the obtained variants increased the light output or shifted the emission peak, yielding RLuc8.6-535, RLuc8.6-545, and RLuc8.6-547, which emit bioluminescence at 535 nm, 545 nm, and 547 nm, respectively. The use of substrate analog coelenterazine-v (Figure 3e) resulted in a further red-shift in RLuc8.6-535 (570 nm) and RLuc8.6-547 (588 nm). In the case of a bioluminescence system composed of CBR2opt as luciferase and NH_2_-NpLH_2_ (Figure 2b) as luciferin, the substrate luciferin was engineered firstly [20]. It has been demonstrated that the extension of the π conjugation in luciferin reduces the HOMO-LUMO energy gap in the light-emitting oxyluciferin, which leads to red-shifted bioluminescence [15,89]. To extend the conjugation, naphthothiazole-based analog was examined as red-shifted substrate luciferin for CBR. Among the candidates, NH_2_-NpLH_2_ and OH-NpLH_2_ (Figure 2c) emitted bioluminescence in the near-infrared region, for which the wavelength reached maximum values at 664 nm and 758 nm, respectively; however, the signal intensities were generally much lower than that of FLuc. To improve the bioluminescence intensity, the putative amino acid residues nearby active pockets (R334 and G351) were subjected to rational site-directed mutation. R334S and G351R mutations with codon optimization generated CBR2opt, which exhibited red-shifted bioluminescence with NH_2_-NpLH_2_ (730 nm) with 2–3 fold more luminescence than FLuc.

The coevolution strategy was also applied to FLuc. Phe247 in the luciferin binding pocket is involved in a π-stacking with d-luciferin, whose F247L mutation lowered the affinity for d-luciferin without impairing catalytic activity [8,12]. The F247L mutant improved the maximal sustained light emission from 6′-aminoluciferin (Figure 2d) by 4.9-fold [13]. In the case of the cyclic version of aminoluciferin called CycLuc1 (599 nm, Figure 2e), the F247A mutation gave a dramatically improved light output. The screening of mutants using CycLuc1 identified several mutants with an improved signal, including R218K, T251S, L286M, and S347A. R218K showed the greatest improvement in CycLuc2 (Figure 2f) emission (607 nm). The further engineering of FLuc and CycLuc identified the pair between FLuc variant (R218K, L286M, and S347A) and CycLuc7 (Figure 2g), which produces red bioluminescence (623 nm) with 46% of the initial rate of the pair between FLuc and d-luciferin [14]. As for CycLuc10 (Figure 2h), the single mutation R218K on FLuc gave the most red-shifted bioluminescence (648 nm). Infra-luciferin (Figure 2i), a d-luciferin derivative with increased conjugation, has an emission peak around 670 nm with wild type FLuc [15]. Further mutations (F14R, L35Q, V182K, I232K, S284T, and F465R) gave more red-shifted bioluminescence at the peak of 706 nm. Infra-luciferin also enabled dual color in vivo BLI using FLuc_green (F14R, L35Q, A105V, V182K, T214C, I232K, D234G, V241I, G246A, F250S, E354R, D357Y, S420T, and F465R) and FLuc_Red (F14R, L35Q, A105V, V182K, T214C, I232K, S284T, D234G, E354I, D357Y, S420T, and F465R) [16]. Another water-soluble luciferin derivative called Akalumine-HCl (Figure 2j) originally showed luminescence reaching a maximum value at 677 nm [17]. Considering that FLuc is not enzymatically optimal for Akalumine-HCl, directed evolution with mutagenesis on luciferase genes, such as FLuc, emerald luciferase, and CBR, was performed [18]. The FLuc-based library produced a variant named AkaLuc, which has 28 amino acid mutations on FLuc. AkaLuc significantly improved the luminescence intensity obtained with Akalumine-HCl 7-fold, with a slight blue-shift (650 nm). The generated bioluminescence system AkaBLI enabled examination of single-cell BLI deep brains of naturally behaving marmosets (Figure 4b). The systematic screening system with molecular designing in silico will become a powerful technique for the further exploration of novel luciferase-luciferin pairs. Such techniques have already been demonstrated by identifying the pair of phenol luciferin (PhOH-Luc, Figure 2k) and FLuc mutant G2 (S220N, E311C, and A313G) that showed a broader emission shoulder in 650–850 nm region [19].

### 3.2. BRET-Based Approaches

The intramolecular BRET-based approach enables the rational design of the red-shifted bioluminescence by fusing the pair of a luminescence donor and fluorescence acceptor. In 1999, the first BRET pair between RLuc and eYFP using coelenterazine (BRET1) was reported, where the emitted light is shifted to 527 nm from 480 nm [27]. The second-generation BRET2 is based on RLuc and GFP2 (GFP variant), which uses DeepBlueC (Figure 3f) as a substrate. BRET2 emits light at 410 nm, which shifts more than 100 nm from the original bioluminescence peak at 395 nm [28]. Based on these findings, the red-shifted bioluminescence system using BRET has been extensively explored. The BRET3 pair between RLuc8 and mOrange shows emission at 564 nm with a 7-fold increase in the intensity compared to BRET1 and BRET2 [29]. Additional BRET pairs have been reported, such as RLuc8 and TagRFP (BRET4), RLuc8.6 and TagRFP (BRET5), or RLuc8.6 and TurboFP635 (BRET6), which emit bioluminescence at 584 nm, 584 nm, or 635 nm, respectively [30,31]. The coelenterazine analog called coelenterazine-v (Figure 3e), which shifts the RLuc8 emission to 515 nm, increased BRET efficiency in the BRET6 pair (BRET6.1), whereas it decreased in the BRET3 pair (BRET3.1). For more red-shifted BRET pairs with RLuc8, BRET pairs with bacterial phytochrome-based NIR fluorescent proteins (iRFPs) were reported [32]. In addition to the absorption peak at 643 nm (iRFP670) or 702 nm (iRFP720), iRFPs have a second absorption peak at around 380 nm called the Soret band, which corresponds to the absorbance of the biliverdin Ivα chromophore [90]. The band made iRFPs favorable acceptors of RLuc8 bioluminescence. Among substrates for these BRET pairs, methoxy-eCoelenterazine (Figure 3g) showed great stability. Because of the 50 nm spectral separation in the emitted luminescence, the BRET pairs enabled multicolor bioluminescence imaging. A recently reported BRET pair between red-shifted variant RLuc8.6-535SG (RLuc8.6-535 with additional S257G mutation) and iRFP with coelenterazine analog BBlue2.3 (Figure 3h) exhibited similar bioluminescence, which reached its maximum value at 717 nm [33]. Color variations for Nano-lantern, where the spatial arrangement of the donor RLuc8∆N3 (S257G) and acceptor Venus∆C10 is optimized, have been reported. The chimeric fusion protein between mTurquoise2∆C10 and RLuc8∆N3 (S257G) emits cyan light (CNL), whereas the one between mKusabiraOrange2 and RLuc8.6-535∆N3 emits orange light (ONL) [35]. Due to their bright bioluminescence, the Nano-lantern color variants enabled multicolor monitoring of subcellular structures, or subcellular Ca^2+^ changes.

NLuc has been also used as a donor luciferase in BRET pairs, which was termed NanoBRET. The intense bioluminescence of NLuc enabled NanoBRET to be used for microscope imaging, such as NanoBRET with YFP for the widefield high-content microscope [36] and NanoBRET with Venus for the inverted microscope [37]. The increased luminescence of NLuc allows energy transfer to a broad range of fluorescence acceptors, enabling improved spectral separation between the donor and acceptor. For example, NanoBRET with a red fluorescent protein mKate2 (ex. 588 nm/ em. 633 nm) was reported [38]. Inspired by the strategy for Nano-lantern development using RLuc, a second-generation Nano-lantern using NLuc was developed by optimizing the spatial orientation:NLuc∆N3 and mTurquoise2∆C10 (CeNL), NLuc∆N5 and mNeonGreen∆C10 (GeNL), NLuc∆N4 and Venus∆C12 (YeNL), and NLuc∆N5 and tdTomato∆C9 (ReNL) [39]. GeNL was recently used for BLI in plant *Arabidopsis thaliana*, which was detected even with the naked eye [91]. In the Nano-lantern using mKOκ (OeNL), mKOκ was inserted into the loop region of NLuc to improve BRET efficiency. Similar molecules called LumiFluors were developed by conjugating NLuc with enhanced GFP (GpNLuc) or with a long Stokes shift mOrange (OgNLuc) [40]. Although both LumiFluors showed equivalent signals in vitro, OgNLuc exhibited a higher signal in vivo, showing that red wavelength emissions allow for greater tissue penetration. An orange-red fluorescent protein CyOFP1, whose emission peak is around 589 nm, acted as a BRET acceptor for NLuc. Fusing two CyOFP1s to both the C- and N-termini of NLuc gave new chimera called Antares, whose BRET signal increased by 2-fold [41]. A recently reported furimazine derivative, hydrofurimazine (Figure 3i) or fluorofurimazine (Figure 3j), which has greater solubility and higher maximum possible dose in vivo, enabled similar or even higher brightness in vivo compared to AkaBLI [42]. To improve the BRET signal, the donor NLuc-furimazine in Antares was replaced with teLuc-diphenylterazine, which overlaps better with the absorbance of CyOFP1 [24]. The yielded protein named Antares2 emits red-shifted bioluminescence at 583 nm, without the loss of brightness in cultured cells, compared to teLuc, which emits bioluminescence at 502 nm. In living mouse organs, on the other hand, Antares2 shows an additional 35–90% signal increase over teLuc, which again shows that the redder one is better for deep tissue BLI. The engineered luciferase LumiLuc improved from teLuc with mutations at E4G, L18Q, S19A, V27L, S28T, G67C, G71A, N85D, V90A, R112Q, V119K, and K136T, was also exploited as a BRET donor [25]. The BRET pair between LumiLuc and mScarlet with 8pyDTZ (Figure 3k) used as a substrate emits bioluminescence around 600 nm. Although the bioluminescence peak is shorter than that of NIR, the bright luminescence of LumiLuc-mScarlet enabled ATP-independent in vivo BLI comparable to that of AkaBLI. Further engineering will be required to achieve adequate substrate biodistribution in living animals, which is essential for in vivo BLI [18].

Other luciferases have been also used as BRET donors. Because FLuc emits bioluminescence around 560–620 nm, red fluorescent proteins are a suitable acceptor in BRET combination, such as DsRed (em. 583 nm) [43], mCherry (em. 610 nm) [44], and mKate (em. 635 nm) [45]. Fluorescence dyes can also be exploited as BRET acceptors. For example, Cy3.5 (ex. 581 nm/ em. 596 nm) was used as the acceptor in BRET using FLuc [46]. To bring dyes in close proximity with luciferases, these reports utilized dye-labeled antibodies. In the BRET pair between the FLuc mutant (PpyRE10: T169C, T214A, A215L, I232A, S284T, F295L, R330G, I351V, S399C, and F465R mutations), which emits red light (617 nm), and Alexa Fluor 680 (AF680) or 750 (AF750), dyes were directly labeled onto the cystine residues of PpyRE10 via the maleimide group of the dyes. They emit bioluminescence at 705 nm (AF680) or 783 nm (AF750) with high BRET efficiency, at the ratio of 34 (AF680) and 4 (AF750) [47]. Reportedly, a novel bioluminescence probe was developed to emit NIR-II light (1029 nm) using direct labeling reactions [92]. The probe, named NIR-II-BP, was generated by loading several fluorescence dyes (Cy5, Cy7.5, and FD-1029) into a self-assembled amphiphilic polymer micelle and by subsequent surface labeling with FLuc. The bioluminescence energy of FLuc is transferred via BRET to Cy5 (ex. 652 nm/ em. 672 nm) [93], and subsequently via FRET to Cy7.5 (ex. 788 nm/ em. 808 nm) [94], and FD-1029 (ex. 977 nm/ em. 1029 nm). The light emitted from NIR-II-BP enabled BLI using the second near-infrared region (1000–1700 nm), which detected tiny lymph node metastases (1–2 mm) with high precision. Self-labeling protein tags are alternative choices for labeling dyes in BRET pairs. SNAP-tag is a protein module that forms covalent bonds with *O*^6^-benzylguanine derivatives [95]. Another protein tag, HaloTag, reacts with chloroalkane linker bound to dyes [96]. Using SNAP-tag or HaloTag7, various fluorescent dyes, including Alexa Fluor 488, TMR, CPY, and SiR were exploited as acceptor fluorophores for BRET with NLuc [48]. Quantum dots, small inorganic semiconductor nanocrystals, were also utilized as acceptors in various BRET pairs. The high extinction coefficients and comparable quantum yields to fluorescent organic dyes make QDs attractive fluorophores. As BRET partners, QD_605_, QD_655_, QD_705_, and QD_800_, have been used with RLuc8 [49,50,51]. The BRET pair between the polymer-coated CdSe/ZnS core–shell QD_705_ and NLuc, which emits strong luminescence at 705 nm, was used for mapping lymph nodes in mouses [52]. The major concern for using QDs in in vivo BLI is the toxicity of inorganic components, such as cadmium. In animal experiments, metal ion release from the surface of QDs leads to their accumulation in the liver, which reduces the viability of hepatocytes [97,98]. Therefore, protective capping of the surface or use of less-toxic components are required to improve biocompatibility in using QDs as BRET acceptors in in vivo BLI.

## 4. Conclusions and Outlook

BLI enables highly sensitive imaging in living organisms because it requires no external light for light emission. However, bioluminescence systems using native luciferases suffered from their weak luminescence intensity or short emission wavelength. The low signal because of their poor tissue penetration hampered precise monitoring of biomolecular reactions at high spatiotemporal resolution. Herein, we explained that considerable efforts have been undertaken using several approaches to develop brighter and red-shifted bioluminescence systems. The bright bioluminescence system NLuc-furimazine and red-shifted AkaLuc/Akalumine-HCl especially have demonstrated excellent performance in diverse BLI trials. Further modulations, including mutagenesis, luciferin engineering, and intramolecular BRET, will engender much brighter and more red-shifted bioluminescence. For the exploration of new luciferase–luciferin pairs, a screening system with molecular designing in silico will become a useful tool [19,99]. The new bioluminescence system will enable unprecedented in vivo BLI in various biological contexts at high spatiotemporal resolutions.

For in vivo BLI, bioavailability of luciferins is also crucially important. Because luciferins are not autonomously synthesized in most model animals, luciferins are exogenously supplied via several injection routes: intravenous injection, intraperitoneal injection, or subcutaneous injection [100]. Although intraperitoneal injection is generally preferred because of its convenience, the inadvertent intra-bowel injection [101], variations in resorption from the peritoneal cavity to bloodstream [102], or preferential distribution near the peritoneum distort the BLI results [103]. On the other hand, intravenous or subcutaneous administration overcomes the near-peritoneal overestimation with improved reproducibility. In addition to the injection routes, their biodistribution is affected by their chemical properties, such as cell-membrane permeability, or by the blood–brain barrier [104,105,106]. As was shown with AkaBLI, molecular engineering on luciferase–luciferin pairs is a reasonable approach to improving substrate biodistribution in model animals [17,18]. Another approach to overcome the problems in luciferin bioavailability is the use of the autonomous bioluminescence system. Reportedly, autonomous bioluminescence systems enabled bioluminescence emission without an exogenous luciferin supply by expressing enzymes involved in luciferin synthesis, which were derived from bacterial or fungal systems [107,108]. The improvement of the luciferin bioavailability in model animals will ensure more reliable results in in vivo BLI.

Over the past decades, the optical control of protein function with optogenetic approaches has gained wide attention because it enables the spatiotemporal perturbation of a specific biomolecular reaction with high precision. The recent advance enabled deep-tissue-penetrating optogenetics by near-infrared light, using state-of-the-art nanotechnologies such as upconversion nanoparticles [109]. Currently, the signal outputs upon stimulation are monitored by fluorescence probes. However, the excitation light required for the indicators is often absorbed or scattered by tissues and overlapped with stimulation light for an optogenetic system. These difficulties hamper precise quantitative analysis of the perturbed biological system. In contrast, bioluminescence indicators require no excitation light. Therefore, they enable simultaneous perturbation and observation of the biomolecular reactions in living organisms. The further refinement of bioluminescent probes with the improved luciferase–luciferin reaction will lead us to a sophisticated bioluminescence measurement and opto-bioanalysis of numerous and diverse complicated biological phenomena using optical perturbation.

## Figures and Tables

**Table 1:** The genetically or chemically engineered bioluminescence system.

Name	Origin	Modification	Luciferin	λ_max_ (nm)	Improvement	Refs.
Mutant E	FLuc	T214A, A215L, I232A, F295L, E345K	d-luciferin	560	27-fold more thermally stable than FLuc	[3]
LGR	FLuc	I423L, D436G, L530R	d-luciferin	560	20-fold lower K_m_ for ATP and d-luciferin, and 4-fold higher *k*_cat_ than FLuc	[3]
YY5	FLuc	T214A, A215L, I232A, F295L, E354K, I423L, D436G, L530R	d-Luciferin	560	14-fold higher *k*_cat_ than FLuc	[3]
x5 FLuc	FLuc	F14R, L35Q, V182K, I232K, and F465R	d-luciferin	554	Enhanced thermostability up to 45 °C with higher pH-tolerance	[4]
x5g	FLuc	F14R, L35Q, V182K, I232K, V241I, G246A, F250S, F465R	d-Luciferin	560	3.69-fold brighter than FLuc	[5]
x12 FLuc	FLuc	F14R, L35Q, A105V, V182K, T214C, I232K, D234G, F295L, E354R, D357Y, S420T, and F465R	d-luciferin	557	Half-life of 15 min at 55 °C	[6]
-	FLuc	R218K	d-luciferin	572		[7]
-	FLuc	R218Q	d-luciferin	608		[7]
-	FLuc	R218A	d-luciferin	611		[7]
-	FLuc	R337K	d-luciferin	595		[7]
-	FLuc	R337Q	d-Luciferin	594		[7]
-	FLuc	H245A	d-luciferin	604		[8]
-	FLuc	G315A	d-Luciferin	607		[8]
-	FLuc	T343A	d-luciferin	617		[8]
-	FLuc	A348V	d-luciferin	610		[8]
-	FLuc	S284T	d-Luciferin	615		[9,10]
Ppy RE9	FLuc	T214A, A215L, I232A, S284T, F295L, I351V, R330G, E354I, and F465R	d-luciferin	617	~11.5% respective light intensities compared to FLuc	[11]
-	FLuc	F247L	6′-aminoluciferin	NA ^a^	F247L induced 4.9-fold increase in light output	[8,12]
-	FLuc	R218K	CycLuc1	609		[13]
-	FLuc	R218K	CycLuc2	621		[13]
-	FLuc	R218K, L286M, and S347A	CycLuc7	623	46% of the initial rate of the pair between FLuc and d-luciferin	[14]
	FLuc	R218K	CycLuc10	648		[14]
-	FLuc	-	Infra-luciferin	670		[15]
-	FLuc	F14R, L35Q, V182K, I232K, S284T and F465R	Infra-luciferin	706		[15]
FLuc_green	FLuc	F14R, L35Q, A105V, V182K, T214C, I232K, D234G, V241I, G246A, F250S, E354R, D357Y, S420T, and F465R	Infra-luciferin	700		[16]
FLuc_red	FLuc	(F14R, L35Q, A105V, V182K, T214C, I232K, S284T, D234G, E354I, D357Y, S420T, and F465R	Infra-luciferin	720		[16]
-	FLuc	-	Akalumine-HCl	677		[17]
AkaLuc	FLuc	T39A, E48Q, I51V, K68R, L86S, Q134R, I136V, Q147R, G175S, N229Y, I231N, F294C, F295L, N308S, H310R, H332R, S347N, I349V, L350M, D357R, A361S, D377V, S456G, N463Y, K524R, L526S, I540T, G545D	Akalumine-HCl	650	Improved luminescence intensity	[18]
Mutant G2	FLuc	S220N, E311C, and A313G	PhOH-Luc	608	Photons in the 650–850 nm region accounted for ∼31% of the total emission	[19]
CBR2opt	CBR	R334S, G351R	d-Luciferin	730		[20]
RLuc8	RLuc	A55T, C124A, S130A, K136R, A143M, M185V, M253L, S287L	Coelenterazine	487	4.3-fold ^a^ brighter than RLuc	[21]
RLuc8.6-535	RLuc	A55T, A123S, C124A, S130A, K136R, A143M, D154M, E155G, D162E, I163L, V185L, M185V, M253L, S287L	Coelenterazine	535 (570 ^b^)	6.0-fold brighter than RLuc	[22]
RLuc8.6-545	RLuc	A55T, A123S, C124A, S130A, K136R, A143M, D154K, E155N, D162E, I163L, M185V, M253L, F261W, S287L	Coelenterazine	545		[22]
RLuc8.6-547	RLuc	A55T, A123S, C124A, S130A, K136R, A143M, D154A, E155G, D162E, I163V, M185V, M253L, F262W, S287L	Coelenterazine	547 (588 ^b^)		[22]
C1A4E	OLuc	A4E, Q11R, A33K, V44I, A54F, P115E, Q124K, Y138I, N166R	Coelenterazine	NA ^a^	88,000-fold brighter than OLuc	[23]
NLuc	OLuc	A4E, Q11R, Q18L, L27V, A33N, K43R, V44I, A54I, F68D, L72Q, M75K, I90V, P115E, Q124K, Y138I, N166R	Furimazine	460	150-fold brighter than either FLuc or RLuc	[23]
teLuc	OLuc	A4E, Q11R, Q18L, D19S, L27V, A33N, K43R, V44I, A54I, F68D, L72Q, M75K, D85N, I90V, P115E, Q124K, Y138I, C164H, N166R	Diphenylterazine	502	2.6-fold brighter than NLuc	[24]
yeLuc	OLuc	F1L, A4E, Q11R, A14D, L27V, D19A, V27L, S28T, A33N, K43R, V44I, A54I, F68D, Q69R, L72Q, M75K, I90V, R112Q, P115E, Q124K, Y138I, L142R, C164S, and N166R	Selenoterazine	527	11.5-fold brighter than NLuc	[24]
LumiLuc	OLuc	A4G, Q11R, D19A, S28T, A33N, K43R, V44I, A54I, G67C, F68D, G71A, L72Q, M75K, I90A, R112Q, P115E, V119K, Q124K, K136T, Y138I, C164H, and N166R	8pyDTZ	525	5-fold brighter than NLuc	[25]

^a^ NA: not available. ^b^ Measured with coelenterazine-v.

**Table 2:** The BRET-based bioluminescence system.

Name	Bioluminescence Donor	Fluorescence Acceptor	Luciferin	λ_max_ (nm)	Note	Refs.
BAF-Y	RLuc	EYFP	coelenterazine	525	7-fold brighter than RLuc	[26]
eBAF-Y	RLuc8	EYFP	coelenterazine	525	25-fold brighter than RLuc	[26]
BRET1	RLuc	eYFP	coelenterazine	527		[27]
BRET2	RLuc	GFP2	DeepBlueC	410		[28]
BRET3	RLuc8	mOrange	coelenterazine	564		[29]
BRET3.1	RLuc8	mOrange	coelenterazine-v	564	Decreased BRET efficiency	[30]
BRET4	RLuc8	TagRFP	coelenterazine	584		[31]
BRET5	RLuc8.6	TagRFP	coelenterazine	584		[30]
BRET6	RLuc8.6	TurboFP	coelenterazine	635		[30]
BRET6.1	RLuc8.6	TurboFP	coelenterazine-v	635	Increased BRET efficiency	[30]
-	RLuc8	iRFP670	methoxy-eCoelenterazine	643		[32]
-	RLuc8	iRFP720	methoxy-eCoelenterazine	702		[32]
iRFP-RLuc8.6-535SG	RLuc8.6-535 (S257G)	iRFP	BBlue2.3	717		[33]
Nano-lantern (YNL)	RLuc8∆N3(S257G)	Venus∆C10	coelenterazine	530	2.9-fold brighter than eBAF-Y	[34]
CNL	RLuc8∆N3 (S257G)	mTurquoise2∆C10	coelenterazine	470		[35]
ONL	RLuc8.6-535∆N3	mKusabiraOrange2	coelenterazine	560		[35]
-	NLuc	YFP	furimazine	530		[36]
-	NLuc	Venus	furimazine	535		[37]
-	NLuc	mKate2	furimazine	633		[38]
CeNL	NLuc∆N3	mTurquoise2∆C10	furimazine	475		[39]
GeNL	NLuc∆N5	mNeonGreen∆C10	furimazine	520		[39]
YeNL	NLuc∆N4	Venus∆C12	furimazine	530		[39]
ReNL	NLuc∆N5	tdTomato∆C9	furimazine	585		[39]
OeNL	NLuc	mKOκ	furimazine	565		[39]
GpNLuc LumiFluor	NLuc	EGFP	furimazine	509	45-fold brighter than Nano-lantern	[40]
OgNLuc LumiFluor	NLuc	LSS mOrange	furimazine	572		[40]
Antares	NLuc	CyOFP1	furimazine, hydrofurimazine, or fluorofurimazine	583	Similar or higher brightness in vivo compared to AkaBLI with hydrofurimazine, or fluorofurimazine	[41,42]
Antares2	teLuc	CyOFP1	diphenylterazine	583	Additional 35–90% signal increase over teLuc,	[24]
-	LumiLuc (teLuc with E4G, L18Q, S19A, V27L, S28T, G67C, G71A, N85D, V90A, R112Q, V119K, and K136T mutations)	mScarlet	8pyDTZ	600		[25]
-	FLuc	DsRed	d-luciferin	583		[43]
-		mCherry	d-luciferin	610		[44]
-		mKate	d-luciferin	635		[45]
-		Cy3.5	d-luciferin	596		[46]
-	Ppy RE10	Alexa Fluor 680	d-luciferin	705	BRET ratio: 34.0	[47]
-		Alexa Fluor 750	d-luciferin	783	BRET ratio: 4.0	[47]
-	NLuc	Alexa Fluor 488	furimazine	525	Via SNAP-tag or HaloTag7	[48]
-		TMR	furimazine	585	Via SNAP-tag or HaloTag7	[48]
-		CPY	furimazine	645	Via SNAP-tag or HaloTag7	[48]
		SiR	furimazine	670	Via SNAP-tag or HaloTag7	[48]
-	RLuc8	QD_605_	coelenterazine	605	BRET ratio: 0.70	[49]
-		QD_655_	coelenterazine	655	BRET ratio: 1.2	[49,50,51]
-		QD_705_	coelenterazine	705	BRET ratio: 2.3	[49]
-		QD_800_	coelenterazine	800	BRET ratio: 1.32	[49]
-	NLuc	QD_705_	furimazine	705	BRET ratio: 13.3	[52]
